# Type 2 Diabetes Modifies Skeletal Muscle Gene Expression Response to Gastric Bypass Surgery

**DOI:** 10.3389/fendo.2021.728593

**Published:** 2021-10-06

**Authors:** Matthew D. Barberio, G. Lynis Dohm, Walter J. Pories, Natalie A. Gadaleta, Joseph A. Houmard, Evan P. Nadler, Monica J. Hubal

**Affiliations:** ^1^ Center for Genetic Medicine Research, Children’s National Research Institute, Washington, DC, United States; ^2^ Department of Exercise and Nutrition Sciences, Milken Institute School of Public Health, George Washington University, Washington, DC, United States; ^3^ Department of Physiology, Brody School of Medicine, East Carolina University, Greenville, NC, United States; ^4^ Department of Surgery, Brody School of Medicine, East Carolina University, Greenville, NC, United States; ^5^ Human Performance Laboratory, Department of Kinesiology, College of Health and Human Performance, East Carolina University, Greenville, NC, United States; ^6^ Division of Pediatric Surgery, Children’s National Hospital, Washington, DC, United States; ^7^ Department of Kinesiology, Indiana University Purdue University Indianapolis, Indianapolis, IN, United States

**Keywords:** skeletal muscle, bariatric (weight-loss) surgery, type 2 diabetes (T2D), gene expression, metabolism

## Abstract

**Introduction:**

Roux-en-Y gastric bypass (RYGB) is an effective treatment for type 2 diabetes mellitus (T2DM) that can result in remission of clinical symptoms, yet mechanisms for improved skeletal muscle health are poorly understood. We sought to define the impact of existing T2DM on RYGB-induced muscle transcriptome changes.

**Methods:**

Vastus lateralis biopsy transcriptomes were generated pre- and 1-year post-RYGB in black adult females with (T2D; *n* = 5, age = 51 ± 6 years, BMI = 53.0 ± 5.8 kg/m^2^) and without (CON; *n* = 7, 43 ± 6 years, 51.0 ± 9.2 kg/m^2^) T2DM. Insulin, glucose, and HOMA-IR were measured in blood at the same time points. ANCOVA detected differentially expressed genes (*p* < 0.01, fold change < |1.2|), which were used to identify enriched biological pathways.

**Results:**

Pre-RYGB, 95 probes were downregulated with T2D including subunits of mitochondrial complex I. Post-RYGB, the T2D group had normalized gene expression when compared to their non-diabetic counterparts with only three probes remaining significantly different. In the T2D, we identified 52 probes upregulated from pre- to post-RYGB, including NDFUB7 and NDFUA1.

**Conclusion:**

Black females with T2DM show extensive downregulation of genes across aerobic metabolism pathways prior to RYGB, which resolves 1 year post-RYGB and is related to improvements in clinical markers. These data support efficacy of RYGB for improving skeletal muscle health, especially in patients with T2DM.

## Introduction

Approximately 9 in 10 individuals with type 2 diabetes mellitus (T2DM) are classified as overweight or obese and display peripheral insulin resistance (1). Roux-en Y gastric bypass (RYGB) weight-loss surgery is recognized as an effective intervention for the treatment and remission of T2DM in individuals with severe obesity (2, 3). As the primary site of glucose disposal in response to acute insulin action (4), skeletal muscle is likely a site of improved metabolic programming in response to intervention such as RYGB surgery (5).

Reduced skeletal muscle mitochondrial content and function in response to nutrient oversupply is a significant modifier of skeletal muscle insulin sensitivity in obesity and T2DM (6). Excess intracellular lipids and dysfunctional insulin receptor signaling leads to blunted expression of mitochondrial genes, including the master mitochondrial biogenesis transcriptional regulator peroxisome proliferator-activated receptor co-activator 1 alpha (PCG1α) (7–10). Improved mitochondrial function has been noted in humans following RYGB (11–13), but a limited number of studies have explored skeletal muscle gene expression profiles as far as 1 year post-surgery (5, 14, 15).

Previous studies exploring human skeletal muscle gene expression profiles following RYGB have identified changes in the expression of genes involved in insulin signaling (6), and inflammation (14), as well as mitochondrial and lipid metabolism (15). However, none of these studies specifically address the presence of T2DM on skeletal muscle gene expression compared to people with obesity but not overt T2DM. Previous studies have largely focused on Caucasian subjects despite black individuals accounting for ~15% of the bariatric surgery population (16). Furthermore, T2DM is more prevalent in black people in comparison to other races, underlining the need to study this population in greater detail to understand potential molecular drivers of this disparity. In the current study, we report the effects of T2DM on vastus lateralis global gene expression profiles prior to and 1 year following RYGB in black women with and without T2DM to determine the modifying effects of existing T2DM in RYGB-response.

## Methods

### Subjects and Clinical Data Collection

Adult black females without (CON; *n* = 7) and with T2DM (T2D; *n* = 5) were recruited from an established bariatric surgery program at Vidant Medical Center (Greenville, NC); all subjects were classified with obesity. Institutional review boards at both East Carolina University and Children’s National Medical Center approved the study and written informed consent was obtained from all study participants. Criteria for inclusion included age between 25 and 60 years, BMI between 35 and 65 kg/m^2^, a negative pregnancy test, and (for T2D group) a diagnosis of T2DM in accordance with the criteria for the NIH Consortium for the Longitudinal Assessment of Bariatric Surgery (17).

Subjects were enrolled in the standard clinical protocol for bariatric surgery at Vidant Health. Anthropometric measures (age, height, weight, and BMI), fasting blood (antecubital) collection, and skeletal muscle biopsies were collected 2 weeks prior to (Pre) and 1-year post-surgery (Post). Whole blood was collected in plasma and serum separating tubes. BMI was calculated as kg/m^2^ and percent excess BMI loss (% Excess BMI Loss) was calculated as [(Pre BMI – Post BMI)/(Pre-BMI – 25)] × 100. Insulin was measured by immunoassay (Access Immunoassay System, Beckman Coulter, Fullerton, CA) and glucose with an oxidation reaction (YSI 2300, Yellow Springs, OH). The homeostasis model assessment (HOMA2) was calculated from plasma glucose and insulin levels (www.dtu.ox.ac.uk/homacalculator) (18).

### Skeletal Muscle Biopsies

Skeletal muscle biopsies were taken 2 weeks prior to and 1 year following RYGB, in a fasted state, from the vastus lateralis muscle of the non-dominant leg *via* standard Bergstrom needle biopsy (19). Approximately 100 to 200 mg of tissue was obtained with a triple pass and immediately flash frozen in liquid nitrogen. Approximately 20 to 30 mg of tissue was used for both RNA and DNA extraction protocols.

### Gene Expression Profiling

Skeletal muscle gene expression was analyzed from skeletal muscle biopsies taken pre- and post-RYGB *via* global microarray analysis (Affymetrix HU133 Plus 2.0 microarray; Affymetrix, Santa Clara, CA; Accession: GSE161643). Total RNA was isolated from skeletal muscle homogenates *via* the TRIzol (Invitrogen. Carlsbad, CA) method (20). Affymetrix instructions were followed for microarray processing. Briefly, 500 ng of total RNA was used with appropriate Poly-A controls for first- and second-strand cDNA synthesis. Biotin-labeled complementary RNA (cRNA) was synthesized using *in vitro* transcription of the second-strand cDNA with a T7 RNA polymerase. Approximately 30 μg of labeled cRNA was fragmented and hybridized to each microarray.

CEL files were generated from scanned microarrays and imported into Affymetrix Expression Console, where CHP files were generated using the PLIER (Probe Logarithmic Intensity Error) algorithm. PLIER is a mode-based signal estimator that takes advantage of numerous internal control probes of the microarray to differentiate between background and signal. Standard quality control methods were used to evaluate amplifications, thresholds for appropriate scaling factors, and RNA integrity (GAPDH 3’/5’ and HSAC07 3’/5’). Samples failing quality standards were reprocessed from original total RNA. Probe lists for statistical analysis (using the PLIER generated probe intensities) were also filtered for present/absent calls using the MAS5.0 algorithm in Expression Console. Probes that were determined present on 20 of 24 arrays (83.3%) were retained for statistical analysis. Resultant CHP files were imported into Partek Genomics Suite (Partek, Inc.; St. Louis, MO). Probe set intensities (PLIER) were log_2_-transformed for data normalization before statistical analyses.

### Differential Expression Analysis and Biological Interpretation of Gene Expression

Differences in gene expression were assessed *via* three-way analysis of covariance using a restricted maximum likelihood approach (Model: time point × group × time point*group + age + BMI) with contrasts between groups and time points conducted *via* Fisher’s least significant difference test (21). Significant probes were defined as *p* < 0.01 and resultant gene sets were uploaded to Ingenuity Pathway Analysis (IPA; Qiagen, Inc.) for probe set annotations and to query relationships between genes. The canonical pathway analysis tool was used to identify biological pathways that were overrepresented in our dataset *via* a right-handed Tukey’s *t*-test (22). We also utilized Gene Ontology (GO) Enrichment Analysis to determine categorization of the biological processes of significant gene lists (23, 24). GO Enrichment Analysis uses a Fisher’s exact test for classification and *p*-values for false discovery rate (FDR) are reported in the results.

### Real-Time PCR Validation of Target Genes

Microarray results were confirmed with real-time polymerase chain reaction (qPCR). Due to RNA quantity and concentrations available following microarray analysis, a representative subset of *n* = 3 in the T2D group was used in qPCR analysis. RNA (100 ng) was reverse-transcribed into cDNA using SuperScript III Reverse Transcription (Invitrogen Corp.; Carlsbad, CA) following manufacturer protocols. PCR was performed in triplicate on an Applied Biosystems QuantStudio 3 Real-Time PCR Systems with Taqman Universal PCR Master Mix and commercially available TaqMan human gene expression assays (ThermoFisher Scientific; Waltham, MA) for hexokinase 2 (HK2; AssayID: Hs00606086_m1), NADH:ubiquinone oxidoreductase subunit B8 (NDUFB8; Hs00428204_m1), NADH:ubiquinone oxidoreductase subunit B7 (NDUFB7; Hs00958815_g1), NADH:ubiquinone oxidoreductase subunit A1 (NDUFA1; Hs00244980_m1), and 3-hydroxybutyrate dehydrogenase, type 1 (BDH1; Hs00366297_m1). Assays were performed in accordance with manufacturer instructions: 50°C for 2 min, 95° for 10 min, followed by 40 cycles of 95°C for 15 s followed by 60°C for 1 min. Assays were run with a multiplexed endogenous control (B2M). Fold changes were determined *via* the 2^−ΔΔCt^ methodology.

### Statistical Analyses

Transcriptome and pathway statistical analyses are described above. Clinical data normality was assessed with Shapiro–Wilk tests and visualization of the distribution. If data were non-normally distributed, the data were log_2_-transformed and reassessed for normality. Differences between groups for anthropometric and clinical data were tested *via* two-sample *t*-test (age, height, % excess BMI Loss, % HOMA-2 Change) and two-way repeated measure ANOVA (group × time × group*time) for remaining measures. When appropriate, a Tukey’s test was used for pairwise comparison. Statistical analyses were performed using OriginLab Pro 2015 (OriginLab Corp, Northampton, MA).

## Results

### Clinical Characteristics

Anthropometric and clinical characteristics are presented in **Table 1**. The T2D group was significantly (*p* = 0.03) older than subjects without diabetes. A time × group interaction (*p* = 0.03) was observed for changes in BMI. Analysis of percent of excess BMI loss indicated that CON lost significantly (*p* = 0.03) more excess BMI as compared to T2D. Blood glucose was significantly (*p* = 0.04) reduced in both groups 1-year following surgery with no significant (*p* = 0.06) difference between groups either pre- or post-surgery. A time × group interaction (*p* < 0.001) was observed for blood insulin. Post-hoc analysis identified that blood insulin was significantly reduced in T2D (*p* = 0.03) and CON (*p* = 0.01) diabetes following surgery. Furthermore, CON had significantly lower blood insulin pre (*p* = 0.007)- and post (*p* = 0.01)-surgery as compared to T2D. Main effects for time (*p* = 0.001) and group (*p* < 0.001) were observed for HOMA-IR, indicating CON had lower HOMA-IR pre- and post-surgery though both groups significantly improved following surgery. However, no difference (*p* = 0.70) was detected between groups for percent change in HOMA-IR following surgery.

**Table 1:** Clinical characteristics.

	CON		T2D	
	Pre	Post	Pre	Post
*N*	7		5	
Age, years	42 ± 6		51 ± 6*	
Height, cm	163 ± 6.0		159 ± 15	
Weight, kg**	135 ± 60	88 ± 27	148 ± 18	113 ± 21
BMI, kg/m^2^***	51.0 ± 9.2	33.3 ± 10.5	52.9 ± 5.8	40.7 ± 6.9
% Excess BMI loss		74.7 ± 32.9*		45.4 ± 15.0
Blood glucose, mg*dl^−1^**	97.8 ± 7.4	86.6 ± 4.4	136.8 ± 54.4	93.7 ± 6.6
Insulin, μU*ml^−1^***	11.6 ± 3.5	3.3 ± 1.3	48.4 ± 23.8	17.3 ± 16.9
HbA1c (%)	5.98 ± 0.3	NA	7.14 ± 1.1	5.46 ± 0.5
HOMA-2☨	1.5 ± 0.5	0.5 ± 0.1	5.6 ± 1.6	2.2 ± 2.1
% HOMA Change	61.4 ± 26.9		55.4 ± 43.3	

Data are presented as Mean ± Std. Dev

*Student’s t-test p < 0.05 as compared CON or T2D.

**Two-way RM ANOVA p < 0.05 for time. No group effect.

***Two-way RM ANOVA p < 0.05 for time × group.

*☨*Two-way RM ANOVA p < 0.05 for time and group. No time × group effect.

NA, Not Available.

### Skeletal Muscle Global Gene Expression

Following preliminary filtering for MAS5.0 present/absent (83.3% cutoff) left 25,839 probes for statistical analysis using PLIER generated probe intensity values. ANCOVA detected 114 significant (*p* < 0.01; **Supplementary Table S1**) probes for the main effect of time point, 61 significant (*p* < 0.01; **Supplementary Table S2**) probes for the main effect of group, and 376 significant (*p* < 0.01; **Supplementary Table S3**) probes for time × group interaction. The 376 significant probes from the time × group interaction were carried forward for pairwise comparisons.

### Pre-Surgery Differences in Skeletal Muscle Oxidative Metabolism Gene Expression

**Figure 1:**
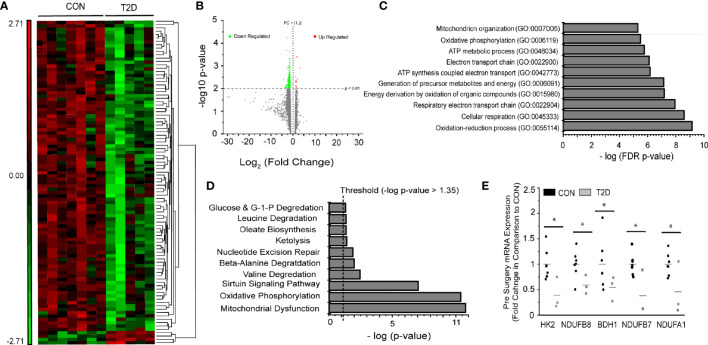
Pre-surgery skeletal muscle gene expression in T2D vs. CON. **(A)** Heat map (probe intensities) of 97 significantly (p < 0.01; FC > |1.2|) probes used for biological function and pathway analysis. **(B)** Volcano plot of 25,839 probes used for statistical analysis. Green (Down Regulated) and Red (Upregulated) of 97 significant (*p* < 0.01, FC > |1.2|) probes for Pre-Surgery skeletal muscle T2D vs. CON. **(C)** Gene Ontology Enrichment analysis for biological process of differentially expressed genes in individuals with and without diabetes pre-surgery. **(D)** Significant pathways from Ingenuity Pathway Analysis using the Canonical Pathway tool. Presented pathways are overrepresented with genes differentially regulated between those with and without diabetes. **(E)** Fold changes in select genes as determined via qPCR; *p < 0.05 students t-test.

Baseline pairwise comparison (Pre-RYGB T2DM vs. Pre-RYGB CON) resulted in 97 significant probes (**Figures 1A, B**, **Supplementary Table S4**), of which 95 had lower expression in T2D. We used multiple bioinformatics knowledge base tools to explore the biological context of our significant probe list. Using Gene Ontology Enrichment Analysis to categorize our probes based on biological processes, 82 of the 97 probes mapped to known genes and 52 of those were categorized under GO:0008152 Metabolic Process (FDR *p* = 1.72 × 10^-2^). **Figure 1B** depicts the most significant Gene Ontology terms from significant genes prior to surgery. IPA identified 11 enriched canonical biological pathways (**Figures 1C, D**), the majority of which are involved in oxidative metabolism. The top canonical pathways identified were “Mitochondrial Dysfunction” (*p* = 1.66 × 10^-11^; 12 genes), “Oxidative Phosphorylation” (*p* = 4.45 × 10^-11^; 10 genes), and “Sirtuin Signaling Pathway” (8.89 × 10^-8^; 11 genes). Genes involved in oxidative metabolism identified *via* Canonical Pathway Analysis in IPA are listed in **Table 2**. Differences in gene expression between groups pre-surgery for five genes (**Figure 1E**) were confirmed *via* qPCR.

**Table 2:** Baseline (Pre-RYGB) group differences in skeletal muscle gene expression for oxidative pathways.

Gene Symbol	Entrez Gene Name	Affymetrix Probe ID	*p*-value	Fold Change
HK2	hexokinase 2	202934_at	0.008	−3.4
SDHC	succinate dehydrogenase complex, subunit C, integral membrane protein, 15kDa	216591_s_at	0.008	−3.1
		202004_x_at	0.002	−1.6
		210131_x_at	0.003	−1.6
		215088_s_at	<0.001	−1.5
FBXO28	F-box protein 28	1555972_s_at	0.009	−3.0
ACOT11	acyl-CoA thioesterase 11	216103_at	0.008	−3.0
BDH1	3-hydroxybutyrate dehydrogenase, type 1	211715_s_at	<0.001	−2.8
CA14	carbonic anhydrase XIV	219464_at	0.007	−2.7
NRG4	neuregulin 4	242426_at	0.007	−2.4
PPP1R16A	protein phosphatase 1, regulatory subunit 16A	225203_at	0.000	−2.3
ERCC8	excision repair cross-complementing rodent repair deficiency, complementation group 8	1554883_a_at	0.006	−2.3
APOE	apolipoprotein E	203381_s_at	0.009	−2.3
ALDH6A1	aldehyde dehydrogenase 6 family, member A1	221590_s_at	0.006	−2.2
ATP5G3	ATP synthase, H+ transporting, mitochondrial Fo complex, subunit C3 (subunit 9)	228168_at	0.001	−2.1
PECR	peroxisomal trans-2-enoyl-CoA reductase	221142_s_at	0.004	−2.0
FAM221A	family with sequence similarity 221, member A	228600_x_at	0.009	−1.9
SIVA1	SIVA1, apoptosis-inducing factor	203489_at	0.003	−1.9
LIN52	lin-52 homolog (C. elegans)	228583_at	0.004	−1.9
IDH1	isocitrate dehydrogenase 1 (NADP+), soluble	201193_at	0.001	−1.8
TMEM120A	transmembrane protein 120A	223482_at	0.008	−1.8
ECHDC1	enoyl CoA hydratase domain containing 1	223088_x_at	0.006	−1.8
SURF4	surfeit 4	222979_s_at	0.004	−1.7
C6orf108	chromosome 6 open reading frame 108	204238_s_at	0.005	−1.7
C12orf60	chromosome 12 open reading frame 60	243056_at	0.008	−1.7
CEP70	centrosomal protein 70kDa	224150_s_at	0.009	−1.7
MPHOSPH6	M-phase phosphoprotein 6	203740_at	0.005	−1.6
PHB	prohibitin	200658_s_at	0.001	−1.6
MRPS12	mitochondrial ribosomal protein S12	204331_s_at	0.003	−1.6
TMEM126A	transmembrane protein 126A	223334_at	0.008	−1.6
ATP5G1	ATP synthase, H+ transporting, mitochondrial Fo complex, subunit C1 (subunit 9)	208972_s_at	<0.001	−1.6
TMEM161B	transmembrane protein 161B	236227_at	0.008	−1.6
NDUFB8	NADH dehydrogenase (ubiquinone) 1 beta subcomplex, 8, 19kDa	201226_at	0.003	−1.6
MRPL11	mitochondrial ribosomal protein L11	219162_s_at	0.007	−1.6
ALKBH7	alkB, alkylation repair homolog 7 (*E. coli*)	223318_s_at	0.008	−1.6
ERCC8	excision repair cross-complementing rodent repair deficiency, complementation group 8	205162_at	0.008	−1.5
TOMM22	translocase of outer mitochondrial membrane 22 homolog	222474_s_at	0.004	−1.5
FASTKD1	FAST kinase domains 1	219002_at	0.007	−1.5
PXMP2	peroxisomal membrane protein 2, 22kDa	219076_s_at	0.008	−1.5
MRPL47	mitochondrial ribosomal protein L47	223480_s_at	0.009	−1.5
CAMTA1	calmodulin binding transcription activator 1	225693_s_at	0.002	−1.5
TRAM2	translocation associated membrane protein 2	1554383_a_at	0.004	−1.5
MINOS1	mitochondrial inner membrane organizing system 1	224867_at	0.001	−1.5
FTH1	ferritin, heavy polypeptide 1	200748_s_at	0.001	−1.5
PKM	pyruvate kinase, muscle	201251_at	0.010	−1.5
C1orf31	chromosome 1 open reading frame 31	225638_at	0.004	−1.5
STAU2	staufen, RNA binding protein, homolog 2 (Drosophila)	204226_at	0.005	−1.5
HIGD1A	HIG1 hypoxia inducible domain family, member 1A	217845_x_at	0.001	−1.5
PRDX5	peroxiredoxin 5	1560587_s_at	0.002	−1.5
SLC25A12	solute carrier family 25 (aspartate/glutamate carrier), member 12	203339_at	0.008	−1.5
NDUFA1	NADH dehydrogenase (ubiquinone) 1 alpha subcomplex, 1, 7.5kDa	202298_at	0.005	−1.4
NDUFB7	NADH dehydrogenase (ubiquinone) 1 beta subcomplex, 7, 18kDa	202839_s_at	0.004	−1.4
NDUFAF2	NADH dehydrogenase (ubiquinone) complex I, assembly factor 2	228355_s_at	0.009	−1.3
NDUFB3	NADH dehydrogenase (ubiquinone) 1 beta subcomplex, 3, 12kDa	203371_s_at	0.009	−1.3

Fold change = pre-surgery expression T2D/pre-surgery expression CON.

### Improvements in Oxidative Metabolism Gene Expression and Resolution of Gene Expression Differences 1-Year Post-Surgery in Individuals With Diabetes

Comparison of skeletal muscle gene expression profiles 1-year post-surgery resulted in only three significant probes that mapped to three genes: ring finger protein 6 (RNF6; *p* = 0.003, FC = 1.8 greater in individuals with diabetes vs. without), coiled-coil domain containing 90A (CCDC90A; *p* = 0.007, FC = 1.3), and guanine monophosphate synthetase (GMPS; *p* = 0.002, FC = −1.3). These data represent a “closing of the baseline gap” between T2D and CON skeletal muscle health post-surgery. Only CCDC90A (*p* = 0.002; FC = −1.2) was found to be significantly different in pre-surgery gene expression analysis.

**Figure 2:**
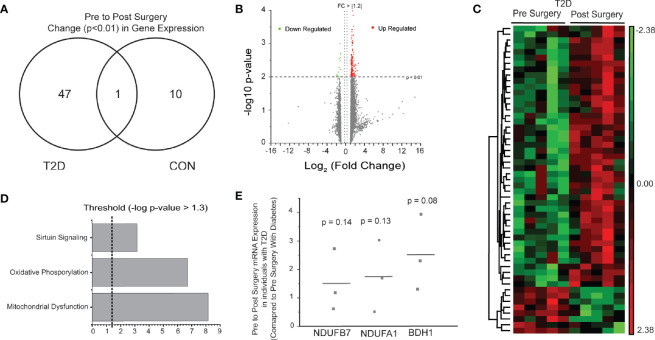
Pre to post-surgery skeletal muscle gene expression. **(A)** Venn diagram of significant probes in individuals with diabetes and without diabetes, and overlap, pre-to-post surgery. **(B)** Volcano plot of 25,839 probes used for statistical analysis. Green (Down Regulated) and Red (Upregulated) of 53 significant (*p* < 0.01, FC > |1.2|) probes for Pre-to-Post surgery gene expression in T2D skeletal muscle. **(C)** Heat map (probe intensities) of 48 significant (*p* < 0.01, FC > |1.2|) probes pre-to-post surgery in individuals with diabetes. **(D)** Significant pathways from Ingenuity Pathway Analysis using the Canonical Pathway tool. Presented pathways are overrepresented with genes differentially regulated between pre-to-post surgery in individuals with diabetes. **(E)** Fold changes in select genes as determined *via* qPCR.

We explored changes in skeletal muscle gene expression profiles pre- to post-surgery in individuals with diabetes and without diabetes (**Figure 2A**). Comparison of pre- to post-surgery gene expression changes identified 53 probes (**Supplementary Table S5**), mapping to 48 known genes. An increase in expression (FC > 1.2) was observed in 43 of the 48 known genes (**Figures 2B, C**). Only 11 genes were significantly altered pre- to post-surgery in individuals without diabetes. Gene Ontology Enrichment Analysis for Biological Process of genes with significant differential expression pre- to post-surgery in individuals with diabetes identified respiratory electron transport chain (FDR = 6.8 × 10^-2^) and oxidative phosphorylation (FDR = 4.18 × 10^-2^) as the only significant biological processes. Similarly, canonical pathway analysis again identified Oxidative Phosphorylation (5.19 × 10^-8^; 6 genes), Mitochondrial Dysfunction (1.15 × 10^-6^; 6 genes), and Sirtuin Signaling Pathway (3.3 × 10^-3^; 4 genes) as the top overrepresented pathways in the gene list (**Figure 2D**). Significant genes pre- to post-surgery involved in oxidative metabolism identified *via* Canonical Pathway Analysis in IPA are listed in **Table 3**. Increased expression of three genes (i.e., fold changes) in individuals with diabetes were confirmed *via* qPCR, but were not statistically different (**Figure 2E**).

**Table 3:** Surgery-responsive changes in genes in oxidative pathways in the T2D group.

Gene Symbol	Entrez Gene Name	Affymetrix Probe ID	*p*-value	Fold Change Pre- to Post-Surgery
ATP5O	ATP synthase, H+ transporting, mitochondrial F1 complex, O subunit	216954_x_at	0.006	1.3
COX6C	cytochrome c oxidase subunit VIc	201754_at	0.005	1.3
NDUFB7	NADH dehydrogenase (ubiquinone) 1 beta subcomplex, 7, 18kDa	202839_s_at	0.002	1.3
UQCR10	ubiquinol-cytochrome c reductase, complex III subunit X	218190_s_at	0.003	1.3
PRDX5	peroxiredoxin 5	222994_at	0.007	1.3
NDUFAF2	NADH dehydrogenase (ubiquinone) complex I, assembly factor 2	228355_s_at	0.007	1.3
TUBA4A	tubulin, alpha 4a	212242_at	0.003	1.4
NDUFA1	NADH dehydrogenase (ubiquinone) 1 alpha subcomplex, 1, 7.5kDa	202298_at	0.006	1.4
ATP5G1	ATP synthase, H+ transporting, mitochondrial Fo complex, subunit C1 (subunit 9)	208972_s_at	<0.001	1.6
BDH1	3-hydroxybutyrate dehydrogenase, type 1	211715_s_at	0.008	1.8

Fold change = post-surgery expression T2D/pre-surgery expression T2D.

## Discussion

The purpose of the present study was to identify the effect of T2DM on skeletal muscle gene expression profiles before and after RYGB surgery in severely obese black women. Our data provide evidence that oxidative metabolism genes in women with T2DM are lower prior to RYGB compared to those without T2D, suggesting more dysfunction with the disease. One year following RYGB, skeletal muscle gene expression profiles of those with diabetes normalized compared to their non-diabetic counterparts, largely driven by significant increases in gene expression of electron transport chain genes. Mitochondrial function and perturbations in oxidative metabolism have been implicated in the development of insulin resistance and T2DM (6, 8, 10). RYGB is an effective strategy for significant reductions in excess weight and BMI, as well as the remission of T2DM, though mechanistic understanding of how this resolution occurs remains a work in progress (2, 3). Taken in conjunction with improvements in cardiometabolic profiles (decreased resting blood glucose, insulin, HOMA-2, weight, and BMI), coordinated increases in skeletal muscle oxidative metabolism gene expression appears to play, in part, a role in the remission of T2DM following RYGB surgery.

### Reduced Expression of Aerobic and Mitochondrial Pathways in Skeletal Muscle of Black Women With T2D

**Figure 3:**
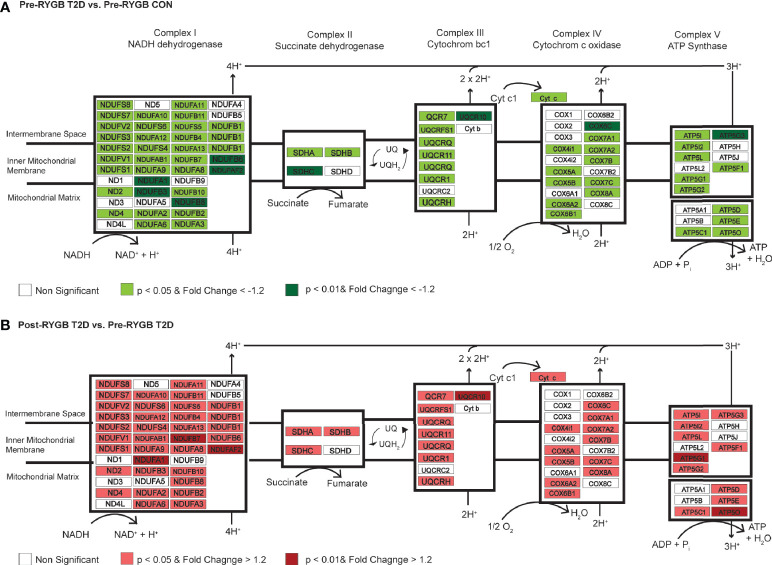
Differential regulation of genes of the mitochondrial complexes between individuals with and without diabetes and pre- to post-surgery in individuals with diabetes. **(A)** Differentially regulated genes in Complex I, II, III, IV, and ATP Synthase in skeletal muscle of individuals with and without diabetes prior to surgery. Using an expanded gene list to include genes with *p* < 0.05, FC > −1.2 (lighter red shading; **Supplementary Table S6**), we show consistent downregulation of genes in mitochondrial complexes in individuals with diabetes prior to surgery. Green shading indicates *p* < 0.01 and FC > −1.2. **(B)** Differentially regulated genes in Complex I, II, III, IV, and ATP Synthase in skeletal muscle of individuals with diabetes pre- to post-surgery. Using an expanded gene list to include genes with *p* < 0.05, FC > −1.2 (lighter green shading; **Supplementary Table 6**), we show consistent upregulation of genes in mitochondrial complexes 1 year post-surgery. Red shading indicates *p* < 0.01 and FC > 1.2.

Individuals with T2DM show significant reductions in skeletal muscle oxidative metabolism gene expression prior to bariatric surgery (15, 25, 26). The current data indicate reductions in multiple subunits of mitochondrial complex I (NDUFB8, NDUFA1, NDUFB7, NDUFAF2, and NDUFB3) in individuals with T2DM prior to RYGB (**Figure 3A**). The role of complex I in the development and treatment of T2DM has been explored in various tissue and cells with conflicting conclusions. Chemical inhibition and gene silencing of complex I shows improved glucose consumption in HepG2 and C2C12 cells as well as improved glucose homeostasis in db/db mice (27) whereas complex I deficits result in metabolic inflexibility in the diabetic heart (28). Most recent evidence suggests that clinical dosage of metformin improves complex I activity (29) through activation of AMPK despite previous reports of supraphysiologic dosages causing inhibition (30). There is also evidence that complex I deficits restrict fetal skeletal muscle growth (31) and decrease mitochondrial efficacy in aging skeletal muscle (32). Expanded analysis (**Supplementary Table S6**) of skeletal muscle gene expression differences in those with and without diabetes prior to surgery shows consistent downregulation of a number of genes for mitochondrial complexes in individuals with diabetes (**Figure 3A**).

We also show concurrent downregulation of key metabolic regulatory genes (**Table 2**) in individuals with diabetes prior to surgery. Hexokinase 2 (HK2; FC = −3.4), succinate dehydrogenase complex, subunit C (SDHC; FC = −3.1), isocitrate dehydrogenase 1 (IDH1; FC = −1.8), and pyruvate kinase muscle (PKM; FC = 1.5) were all downregulated in individuals with diabetes. Taken in conjunction with decreased gene expression of the mitochondrial complexes, black women with T2DM show coordinated downregulation of aerobic metabolism genes in comparison to their non-diabetic counterparts.

### RYGB Normalizes Skeletal Muscle Gene Expression Profiles of Black Women With Diabetes

One year following RYGB, the T2D group had significant improvement in clinical profiles similar to CON: normalized glycemia, reduced hyperinsulinemia, improved HOMA-IR, and significant reduction in excess weight and BMI. Comparison of skeletal muscle gene expression profiles 1 year following RYGB surgery between those with and without diabetes resulted in only three differentially regulated probes between the groups. This indicates that RYGB, and the following lifestyle changes not accounted for here, resulted in skeletal muscle gene expression of individuals with diabetes normalizing to their non-diabetic counterparts. This occurred despite the individuals with T2D being slightly older, suggesting that this age difference was likely not responsible for the initial differences in gene expression. Similarly, the normalization indicates the robust effects of the surgery/weight loss. Further comparison of skeletal muscle expression profiles pre- to post-RYGB in individuals with T2DM showed that almost all (43 of 48) differentially regulated genes increased following surgery and biological interpretation of these genes identified their role in mitochondrial function and aerobic metabolism.

We identified significant upregulation (**Table 3**) of mitochondrial complex I genes (NDUFB8, NDUFAF2, and NDUFA1), ATP synthase subunits (ATP5O and ATP5G1), and cytochrome c oxidase subunit Vic (COX6C) in individuals with T2DM 1 year post-RYGB surgery. Skeletal muscle ETC protein content has been shown to be progressively diminished, including reductions in COX6C, in nondiabetic individuals with obesity and individuals with T2DM in comparison to lean counterparts (33). The degree to which these mitochondrial gene expression changes drive improved clinical phenotypes is indeterminable, but even changes in a small number of genes can have cascade-like effects on cellular signaling and function. For instance, they may be heavily involved in promoting epigenetic (e.g., DNA methylation) changes in muscle (15, 34). Gene expression changes 52 weeks following surgery was more strongly linked to epigenetic (DNA methylation) changes in skeletal muscle than 2 weeks post-surgery when insulin resistance had already resolved (15). Barres et al. (34) also showed that changes in expression of three genes was linked to changes in over 100 CpG methylation sites. Thus, the interpretation that gene expression changes in aerobic metabolism and mitochondrial function genes cannot be limited to improved mitochondrial function or flexibility driving improved phenotype (e.g., resolution of insulin resistance or T2DM). As with our pre-surgery analysis, expanded analysis (*p* < 0.05 and FC = |1.2|; **Supplementary Table S6**) shows consistent upregulation of a number of genes in all mitochondrial complexes 1 year following surgery in those with diabetes (**Figure 3B**).

### Expression of 3-Hydroxybutyrate Dehydrogenase, Type 1 is Reduced in Skeletal Muscle of Black Women With T2D and Improved Following RYGB

Ketone bodies are important metabolic fuels produced in the liver and metabolized in the mitochondria of non-hepatic tissues (35). In our analysis, we show reduced expression of 3-hydroxybutyrate dehydrogenase, type (BDH1), an important catalyst of ketone metabolism, in skeletal muscle of T2D (**Table 2**), which was significantly upregulated 1 year following RYGB (**Table 3**). Expression of BDH1 in adipose tissue has been positively correlated to insulin sensitivity in ~9,000 Finnish men (36), but not associated with circulating ketone levels. Expression of BDH1 in skeletal muscle is modulated by PGC-1α (35), but its potential as a significant modifier of skeletal muscle metabolic function following surgery has not been explored to date. Given the role of β-hydroxybutyrate, the primary circulating ketone body, as a metabolic intermediate and its potential epigenetic signaling actions (37), more exploration into this gene and protein in the context of weight loss surgery is warranted.

## Conclusion

In the present study, we explored skeletal muscle gene expression profiles of black women with and without T2DM prior to and 1 year following RYGB. The presence of T2DM resulted in coordinated downregulation of genes in aerobic metabolism and oxidative metabolic pathway, specifically, subunits for mitochondrial complex I and complex II, as well as other key metabolic regulatory genes. One year following surgery, skeletal muscle gene expression profiles of black women with T2DM had normalized to their non-diabetic counterparts, driven largely by improvements in genes involved in aerobic metabolism and mitochondrial function. Weight loss surgery is an incredibly effective treatment for weight loss and remission for T2DM, and changes in skeletal muscle gene expression may, in part, contribute to these phenotypic changes through improved metabolic and mitochondrial function as well as other cellular signaling functions such as epigenetic modifications. These genes and proteins should be further explored in the context of surgical weight loss and other lifestyle modification strategies to understand their role throughout the dynamic process of severe weight loss and improved metabolic phenotype.

## Data Availability Statement

The datasets presented in this study can be found in online repositories. The names of the repository/repositories and accession number(s) can be found in the article/**Supplementary Material**.

## Ethics Statement

The studies involving human participants were reviewed and approved by East Carolina University and Children’s National Medical Center. The patients/participants provided their written informed consent to participate in this study.

## Author Contributions

MB and MH were involved in all aspects of this study and manuscript preparation. GD, WP, and JH were involved in study design, sample collection, analysis of results, and manuscript preparation. NG was involved in sample processing, data analysis, analysis of results, and manuscript preparation. EN was involved study design, analysis of results, and manuscript preparation. All authors contributed to the article and approved the submitted version

## Funding

This project was supported by Award Number UL1TR000075 (MH) from the NIH National Center for Advancing Translational Sciences and T32AR065993 (MB) from the National Institute of arthritis and Musculoskeletal and Skin Diseases.

## Conflict of Interest

The authors declare that the research was conducted in the absence of any commercial or financial relationships that could be construed as a potential conflict of interest.

## Publisher’s Note

All claims expressed in this article are solely those of the authors and do not necessarily represent those of their affiliated organizations, or those of the publisher, the editors and the reviewers. Any product that may be evaluated in this article, or claim that may be made by its manufacturer, is not guaranteed or endorsed by the publisher.

## Supplementary Material

Supplementary Table 1 Probes and statistics for the main effect of timepoint.

Supplementary Table 2 Probes and statistics for the main effect of group.

Supplementary Table 3 Probes and statistics for time x group interaction.

Supplementary Table 4 Probes and statistics for baseline pairwise comparison (Pre-RYGB T2DM vs Pre-RYGB CON) of skeletal muscle gene expression.

Supplementary Table 5 Probes and statistics for Pre-to-Post surgery skeletal muscle gene expression changes.

Supplementary Table 6 Probes and statistics for expanded gene lists (p < 0.05) used biological pathway analysis.
